# The emerging roles and therapeutic potential of cyclin-dependent kinase 11 (CDK11) in human cancer

**DOI:** 10.18632/oncotarget.8519

**Published:** 2016-03-31

**Authors:** Yubing Zhou, Jacson K. Shen, Francis J. Hornicek, Quancheng Kan, Zhenfeng Duan

**Affiliations:** ^1^ Department of Pharmacy, The First Affiliated Hospital of Zhengzhou University, Zhengzhou, Henan, People's Republic of China; ^2^ Sarcoma Biology Laboratory, Center for Sarcoma and Connective Tissue Oncology, Massachusetts General Hospital, Boston, MA, United States of America

**Keywords:** CDK11, CDKs inhibitor, cell cycle, therapeutic target, cancer therapy

## Abstract

Overexpression and/or hyperactivation of cyclin-dependent kinases (CDKs) are common features of most cancer types. CDKs have been shown to play important roles in tumor cell proliferation and growth by controlling cell cycle, transcription, and RNA splicing. CDK4/6 inhibitor palbociclib has been recently approved by the FDA for the treatment of breast cancer. CDK11 is a serine/threonine protein kinase in the CDK family and recent studies have shown that CDK11 also plays critical roles in cancer cell growth and proliferation. A variety of genetic and epigenetic events may cause universal overexpression of CDK11 in human cancers. Inhibition of CDK11 has been shown to lead to cancer cell death and apoptosis. Significant evidence has suggested that CDK11 may be a novel and promising therapeutic target for the treatment of cancers. This review will focus on the emerging roles of CDK11 in human cancers, and provide a proof-of-principle for continued efforts toward targeting CDK11 for effective cancer treatment.

## INTRODUCTION

Malignant diseases are characterized by uncontrolled cell proliferation and growth [1]. Currently, surgery, radiotherapy, and chemotherapy are still the main modalities in the treatment of cancer [2–4]. Chemotherapy, as one of the basic treatment strategies, is applied in most cancer therapies. However, cancers usually have innate or acquired ability to develop resistance to many anti-cancer drugs, especially in the late stages [5, 6]. Additionally, most conventional chemotherapy drugs applied in the clinic are non-specific cytotoxic agents and will cause serious adverse effects to patients, thus identifying and developing novel therapeutic strategies is an urgent need for cancer treatment.

Cyclin-dependent kinases (CDKs) are serine/threonine kinases that play a critical role in the regulation of cell cycle progression, as well as cellular transcription. Distinct CDKs are activated upon binding with their corresponding cyclin partners. Each kinase in this family is coordinated in an orchestrated way, and responsible for particular aspects of the cellular events. Aberrant expression or altered activity of distinct CDK complexes results in escape of cells from the cell cycle control and leads to malignant transformation. Therefore, the inhibition of CDKs in malignant cells provides a promising approach in the defense against cancer. Recently, many selective CDK inhibitors targeting specific CDKs were developed, which represent promising anti-cancer drugs due to their strong anti-proliferative efficacy combined with a relative low direct cytotoxicity [7–14]. Notably, palbociclib (IBRANCE^®^), a dual CDK4/6 inhibitor, recently received accelerated approval by the Food and Drug Administration (FDA) for clinical breast cancer treatment due to its potent and selective inhibitory effect on estrogen receptor (ER) positive/human epidermal growth factor receptor 2 (HER2) negative breast cancer [15–18]. These developments suggest a promising application of CDK inhibitors as a novel therapeutic strategy in the treatment of human cancer.

CDK11 is a serine/threonine protein kinase in the CDK family. CDK11 also plays a crucial role in cancer cell proliferation and growth. Recent studies have found that the overexpression and activation of CDK11 is crucial in the growth and proliferation of cancer cells, including breast cancer, multiple myeloma, osteosarcoma, and other types of cancer, which has suggested that CDK11 may be a novel potential therapeutic target [19–22]. In this review, we discuss the specificity of CDK11 functions, regulation, and interactions in cancers, as well as the potential of targeting CDK11 in cancer treatment.

## THE BIOLOGY AND DEREGULATION OF CDKS IN HUMAN CANCERS

CDKs are a family of serine/threonine (Ser/Thr) protein kinases, composed of a catalytic kinase subunit and a regulatory cyclin subunit, which play crucial roles in cell cycle progression and transcriptional regulation in response to extracellular and intracellular signals [23]. CDKs are characterized by requirement of protein cyclin subunits for enzymatic activity, even though some CDKs can also play other roles without the involvement of cyclins [24]. Until now, based on the sequence similarity and nomenclature, the human CDKs family include members from CDK1 to CDK20 with specific or redundant roles in many aspects of cell growth and proliferation [25]. The evolutionary relationships among these CDK subfamilies have been identified [26].

Generally, CDKs are divided into cell cycle-related subfamilies and transcription-related subfamilies in regard to their sequencing and main functional roles [26]. Classical cell cycle CDKs, including CDK1, CDK2, CDK4, and CDK6, mainly regulate the transitions between different phases of the cell cycle [27–29]. The cyclin C-CDK3 complex helps the cells to efficiently exit the G0 state and enter the G1 phase. In addition, CDK5, CDK11, CDK14, CDK15, CDK16, CDK17, and CDK18 also belong to the cell cycle-related CDK subfamilies, which play different functional roles by regulating diverse cell cycle progress [30–38] (Table 1). On the other hand, transcription-related CDKs subfamilies function mainly *via* influencing transcription by phosphorylating the carboxy-terminal domain (CTD) of RNA polymerase II (RNAP II), which contains 52 tandem repeats of the consensus heptapeptide amino acid sequence (YSPTSPS) [39, 40]. Specifically, the transcription-related CDKs subfamilies are comprised of CDK7, CDK8, CDK9, CDK11, CDK12, CDK13, CDK19, and CDK20, which participate in different transcription regulation and exert diverse cellular functions [27, 39–49] (Table 1).

**Table 1:** Members of CDK family and their functions in cancers

CDK Family	Cyclins and Cyclin-like Partners	Gene Locus	Functions in Cancers	Inhibitors
**CDK1**	A1, A2, B1, B2, (B3), D, E	10q21.2	promotes cancer cell cycle G2/M transition and proliferation	Pirarubicin, Flavopiridol, Dinaciclib, Seliciclib, Roscovitine, Milciclib, Roniciclib, AZ703, UCN-01, P276-00, AT7519, AZD5438, SCH727965, RGB-286638
**CDK2**	A1, A2, B1, B3, D, E1, E2, Cables1, SpdYA, SpdYC	12q13.2	promotes cancer cell cycle G1/S transition and proliferation	Seliciclib, Flavopiridol, Roscovitine, MLN4924, Dinaciclib, Roniciclib, AZ703, UCN-01, SNS-032, AT7519, SCH727965, RGB-286638, AZD5438
**CDK3**	A1, A2, E1, E2, C, Cables1	17q25.1	helps cancer cells to efficiently exit the G0 state and enter the G1 phase, facilitates cell proliferation	TG-02, AT-7519, RGB-286638
**CDK4**	D1, D2, D3	12q14.1	promotes cancer cell G1 phase progression and proliferation	Palbociclib, Flavopiridol, Abemaciclib, Dinaciclib, Ribociclib, Milciclib, Roniciclib, P276-00, LY2835219, AT7519, MM-D37K, RGB-286638, AZD5438
**CDK5**	p35, p39 (D-, E and G-type cyclins), Cables1	7q36.1	unknown	Roscovitine, Flavopiridol, Milciclib, SCH727965, AZD5438, RGB-286638
**CDK6**	D1, D2, D3	7q21.2	promotes cancer cell G1 phase progression and proliferation	Palbociclib, Flavopiridol, Abemaciclib, Ribociclib, LY2835219, AT7519, RGB-286638
**CDK7**	H	5q13.2	promotes cell cycle progress, RNA transcription, and cancer cell proliferation	Roscovitine, Flavopiridol, Roscovitine, Milciclib, Roniciclib, SNS-032, AT7519, RGB-286638, AZD5438
**CDK8**	C, (K)	13q12.13	activates RNA transcription and promotes cancer cell proliferation	unknown
**CDK9**	K, T1, T2	9q34.11	promotes RNA transcription elongation and cancer cell proliferation	Flavopiridol, Roscovitine, Roniciclib, SNS-032, AZD5438, P276-00, AT7519, SCH727965, RGB-286638
**CDK10**	M	16q24.3	promotes RNA transcription and cancer cell proliferation	unknown
**CDK11**	D3, L1, L2	1p36.33	transcription, RNAsplicing; cell cycle: G2/M	unknown
**CDK12**	L1, L2, K	17q12	promotes RNA transcription elongation, splicing, and cancer cell proliferation	unknown
**CDK13**	L1, L2, K	7p14.1	promotes RNA transcription elongation, splicing, and cancer cell proliferation	unknown
**CDK14**	D3, Y	7q21.13	links cell cycle regulators and Wnt signaling, promotes cancer cell proliferation, migration, and invasion	unknown
**CDK15**	unknown	2q33.1	promotes cancer cell cycle progression and proliferation	unknown
**CDK16**	Y, p35, Cables1	Xp11.3	promotes cancer cell cycle progression and proliferation	unknown
**CDK17**	Cables1	12q23.1	unknown	unknown
**CDK18**	K, A	1q32.1	unknown	unknown
**CDK19**	C	6q21	activates RNA transcription and promotes cancer cell proliferation	unknown
**CDK20**	H	9q22.1	unknown	Unknown

In malignant cells, altered expression of CDKs and their modulators, including overexpression of cyclins and loss of expression of CDK inhibitors, results in deregulated CDK activity, providing a selective growth advantage. In human cancers, owing to various genetic and epigenetic events, CDKs are often overexpressed and/or overactive, bringing about loss of checkpoint integrity and ultimately resulting in uncontrolled cell proliferation [27, 50–53]. For example, the cyclin D/CDK4/CDK6/RB pathway is hyperactive in various malignancies, such as melanoma, glioblastoma, osteosarcoma, lymphomas, breast and cervical cancers, squamous cell cancer, etc [54]. In the transcriptional CDK subfamily CDK9, aberrant activation has also been observed in several primary tumors, including myeloma, prostate cancer, and lung cancer [55].

Because of their critical roles in cell cycle progression and cellular transcription, as well as in the deregulation of human cancer, CDKs comprise an attractive set of targets for novel anticancer drug development [27]. The best known example is the recently FDA-approved CDK4/6 inhibitor palbociclib as the initial endocrine-based therapy for postmenopausal women with ER+/HER2- metastatic breast cancer. Among other members of the CDK family, CDK11 is involved in both cell cycle control and RNA transcription regulation. CDK11 is functionally relevant to many biologic processes, such as RNA transcription and splicing, mitosis, autophagy, and apoptosis [38, 45, 47, 56, 57]. Recently, distinct and unique biologic roles of CDK11 have been discovered in human cancers and in other human diseases [58].

## DISTINCT SPECIFICITY OF CDK11

Unlike other CDKs encoded only by a single gene, CDK11, formerly known as PITSLRE, is encoded by two highly homologous genes, *CDC2L1* (also known as CDK11B) and *CDC2L2* (also known as CDK11A, nonexistent in mouse) in humans. These two genes are localized in a genomic region that spans about 140 kb on human chromosome 1 band p36.3 [59]. In mouse, there is only one gene encoding CDK11 [25]. In human, both of the *CDC2L* genes contain 20 exons and 19 introns that encode almost identical protein kinases named CDK11A and CDK11B.

**Figure 1:**
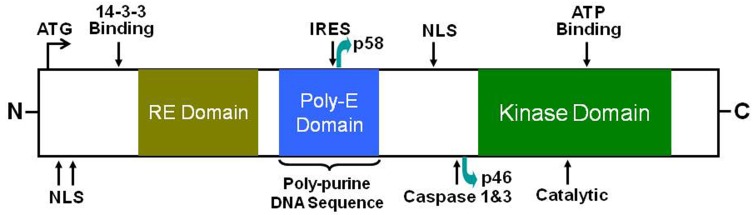
Schematic diagram of the full length CDK11 protein kinase CDK11 is composed of an N-terminal regulatory region, which has multiple nuclear localization signals (NLS) and a 14-3-3 consensus site, and a carboxy-terminal (C-terminal) catalytic domain that is responsible for its kinase activity. There are two separate domains, an RE domain and a poly-E domain located in the center of the CDK11 protein. The full-length CDK11^p110^ isoform contains an IRES and a caspase-3 site, which leads to the generation of a larger CDK11^p58^ and a smaller CDK11^p46^ isoform, respectively (adapted from Trembley et. al., 2004.). NLS, nuclear localization signal; RE, arginine (R) and glutamic (E) acid residues; IRES, internal ribosomal entry site.

CDK11 is composed of an N-terminal regulatory region, which has multiple nuclear localization signals (NLS) and a 14-3-3 consensus site, and a carboxy-terminal (C-terminal) catalytic domain that is responsible for its kinase activity [40, 60]. There are two separate domains, an arginine/glutamic acid domain (RE domain) and a poly-glutamic acid domain (poly-E domain) located in the center of the CDK11 protein (Figure 1) [40]. The RE domains are linked to association with RNA processing factors and poly-E domains are emerging as potential cytoskeletal interacting domains that support RE domain function and aide in keeping these proteins subnuclear. The most important conserved amino acids in CDK11 are the PSTAIRE-helix and three phosphorylation sites, which are involved in the activation and repression of CDK kinase activity [40].

CDK11 binds to L-type cyclins and participates in the coordination between transcription and RNA processing, particularly alternative splicing [61]. The functions of CDK11 have been proved to be linked with RNA transcription and processing, regulation of cell cycle, neuronal function, and apoptosis [38, 40, 47, 56, 58]. The potential for CDK11 to regulate these diverse cellular activities is unique in the CDK family and highlights that CDK11 may exert critical regulatory roles in human tumorigenesis and malignant characteristics of cancer cells.

## DIFFERENT ISOFORMS OF CDK11

Due to the distinct structure and alterative RNA splicing, the *CDC2L* gene can produce three different CDK11 isoforms, a larger 110 kDa protein isoform, a mitosis-specific 58 kDa isoform, and a smaller apoptosis-specific 46 kDa isoform (Table 2). The larger CDK11^p110^ isoform is coded by the full-length CDK11 mRNA and contains an internal ribosome entry site (IRES), which leads to the generation of the CDK11^p58^ isoform during the G2/M phase of the cell cycle. In response to apoptotic signaling, both CDK11^p110^ and CDK11^p58^ isoforms can be cleaved by caspases 1 and 3 and produce the smaller CDK11^p46^ isoform (Figure 1) [62–64]. These different protein kinase isoforms play diverse cellular functions, including RNA transcription and processing, mitosis, and apoptosis. The larger CDK11^p110^ kinase is ubiquitously and constantly expressed throughout the cell cycle. Using subcellular fractionation techniques, the CDK11^p110^ isoform is proven to be a nuclear protein, which localizes to both splicing factor compartments and to the nucleoplasm [65]. On the other hand, the CDK11^p58^ protein is specifically translated from an internal ribosome entry site and expressed transiently only in the G2/M phase of the cell cycle [66]. Due to the fact that CDK11^p58^ is produced during a very narrow window of mitosis, it is much more difficult to detect than CDK11^p110^; its detection depends primarily on the mitotic characteristics of a particular cell type [40, 66]. Although CDK11^p58^ shares the same sequences, including the kinase domain at the C-terminus of CDK11^p110^, the two isoforms possess different functions. The CDK11^p110^ isoform is mainly associated with RNA transcription and splicing [40, 45, 60, 61, 67–69], while CDK11^p58^ isoform is involved in mitosis [70–77]. In contrast to CDK11^p110^ and CDK11^p58^, CDK11^p46^ localizes to the cytoplasm when ectopically expressed. Like other CDKs, the activation of CDK11 also needs to binding with special cyclins. Cyclins that bind CDK11^p110^, CDK11^p58^, and CDK11^p46^ are L-type cyclins, which are encoded by two cyclin L genes encoding a total of six isoforms [61]. CDK11^p58^ also binds cyclin D3 [78]. Several non-cyclin partners have also been proposed to interact with CDK11^p46^, such as eukaryotic initiation factor 3 p47 protein (eIF3 p47) and Ran-binding protein (RanBPM) [79, 80].

**Table 2:** Biological characteristics of three main CDK11 isoforms

CDK11 isoforms	Molecular Weight	Cyclin Partners	Subcellular Localization	Cell Cycle Expression	Expression in Cancer	Cellular Functions
**CDK11^p110^**	110 kDa	cyclins L1 and L2	nucleus	throughout cell cycle	highly expressed	transcription RNA splicing hedgehog signaling pathway Wnt/β-catenin signaling pathway
**CDK11^p58^**	58 kDa	cyclins L1, L2 and D3	nucleus and cytoplasm	transiently in G2/M phase	unknown	mitosis process centrosome maturation bipolar spindle assembly
**CDK11^p46^**	46 kDa	cyclins L1 and L2	cytoplasm	transiently	unknown	apoptosis autophagy

## FUNCTIONS OF CDK11 IN NORMAL CELLS

The CDK11 null cells exhibit proliferative defects, mitotic arrest, and apoptosis, thus suggesting that CDK11 kinase is critical for embryonic development and cellular viability [73]. CDK11 knockout mice display an earlier lethality phenotype during the blastocyst stage of embryonic development. The larger CDK11^p110^ isoform, ubiquitously and constantly expressed throughout the cell cycle, is implicated in pre-mRNA splicing and transcription regulation by interacting with numerous proteins involved in the production of RNA transcripts during proliferation, including the largest subunit of RNA polymerase II and casein kinase II [81]. In addition, CDK11^p110^ has also been identified as a positive regulator of hedgehog signaling pathway [82, 83] as well as a modulator of the Wnt/β-catenin signaling cascade [84], both of which play vital roles in the regulation of embryonic development and adult homeostasis [85–87].

## UPSTREAM AND DOWNSTREAM REGULATIONS IN CDK11 EXPRESSION AND FUNCTIONS

The upstream regulation of CDK11 expression has been studied to identify and characterize the CDK11 promoters. As described above, CDK11 is encoded by two highly homologous genes known as *CDC2L1* and *CDC2L2*. The *CDC2L1* gene is regulated by a basal promoter region that is between nucleotides −152 and +11 of the 5′ region of the *CDC2L1* gene while a region between nucleotides −145 and +10 of the 5′ region of *CDC2L2* is confirmed to be critical for basal transcription of the *CDC2L2* gene [88, 89]. In the promoter of the *CDC2L1* gene, there are transcription factor binding sites for Ets-1 and Skn-1 that are necessary for *CDC2L1* gene expression [88]. However, *CDC2L2* gene expression is mainly regulated by Ets-1 and CREB [89].

Checkpoint kinase 2 (CHK2) and casein kinase 2 (CK2) are confirmed to be the upstream regulators in the CDK11 signaling pathway, which interact with CDK11 *via* phosphorylating the serine 737 and serine 227 sites, respectively [67, 81, 90].CDK11 exerts its functional roles by regulating the expression and/or functions of downstream genes. However, the specific downstream signaling pathway mediated by CDK11 has not been fully documented. Interestingly, a kinase siRNA screen identified CDK11 as a crucial regulator of the Hedgehog pathway [83]. CDK11 was confirmed to directly participate in the Hedgehog signaling pathway by functioning downstream of Smo and upstream of the Glioma-associated (Gli) transcription factors. More specifically, CDK11 interacts with the negative regulator Suppressor of Fused (Sufu) protein and relieves its inhibition of Gli, thus activating the Hedgehog signaling pathway, which is associated with developmental abnormalities and cancer [91, 92]. In addition, CDK11 has also been proved to be a positive modulator of the Wnt/β-catenin signaling cascade, whose dysregulation contributes to the development of cancer, using a kinase-targeted high-throughput siRNA screen [84]. Nevertheless, the molecular mechanisms on which CDK11 regulate the Wnt/β-catenin signaling pathway remain unclear.

## CDK11 INTERACTING PROTEINS AND THE ROLES OF CDK11 IN TRANSCRIPTION AND RNA PROCESSING

The transcription process of mRNA requires the interaction of a set of general transcription factors and a wide range of gene-specific factors. The mediator complex is one type of large protein complexes, which play critical roles in RNA production and processing. Human mediator complex consists of RNAP II and several positive and negative transcription and RNA processing factors. CDK11 is proven to be a crucial part of the mediator complex. CDK11/cyclin L controls the assembly of the RNAP II mediator complex in fission yeast [69]. To be note, the absence of two conserved domains in the fission yeast CDK11 and cyclin orthologs and the reported essential roles of some CDK11 isoforms in unrelated processes in metazoans propose that CDK11 is essential for human beings gene transcription and processing [71, 74, 76].

In the regulation of RNA production, CDK11 could directly phosphorylate the CTD of RNAP II and regulates transcription initiation and elongation as well as RNA processing. In addition, CDK11 also exert its roles in the regulation of transcription and/or transcript processing *via* interacting with numerous transcription regulators and RNA splicing factors.

**Figure 2:**
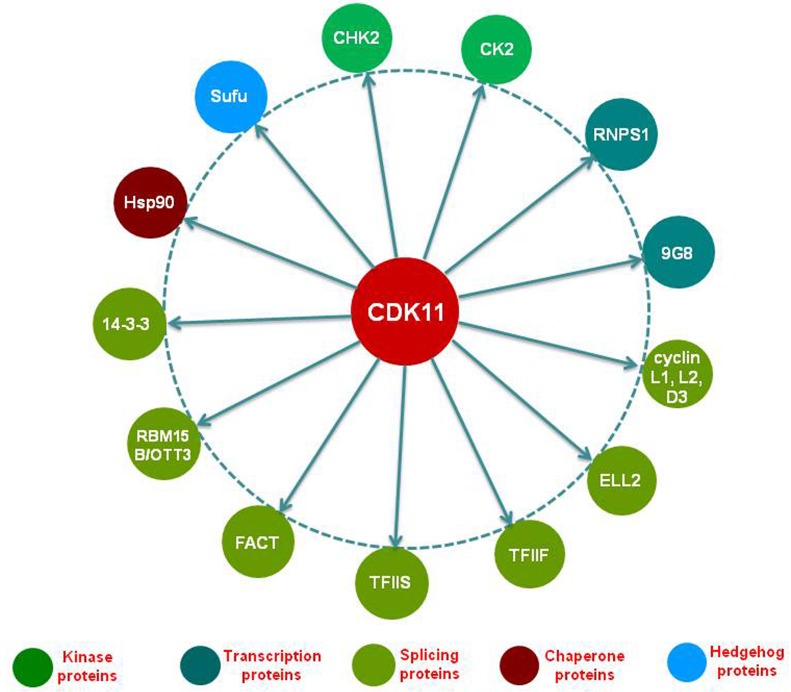
Interacting proteins with CDK11 in transcription and RNA processing The currently identified CDK11 interacting proteins in the transcription and RNA processing are illustrated in this figure. Among which, the protein kinases CHK2 and CK2 activate CDK11 by phosphorylating the serine 737 and serine 227 sites of the CDK11 kinase, respectively. Cyclin L acts as a crucial protein partner of the CDK11^p110^ isoform, while cyclin D3 is essential for functioning for the CDK11^p58^ isoform. RNPSI, 9G8, ELL2, TFIIF, TFIIS, FACT, 14-3-3, and Hsp90 interact with and are subsequently phosphorylated by CDK11. On the other hand, RBM15B/OTT3 interacts with CDK11 as a competitor and antagonizes the positive effect of CDK11 in RNA spicing. Sufu can be negatively regulated by CDK11 and relieve its inhibition on Gli, thus activating the Hedgehog signaling pathway. CHK2, checkpoint kinase 2; CK2, casein kinase 2; RNPS1, RNA-binding protein with serine-rich domain; ELL2, RNA polymerase elongation factor 2; TFIIF, transcription factor IIF; TFIIS, transcription factor IIS; FACT, facilitates chromatin transcription; RBM15B/OTT3, RNA binding motif protein 15 B; Hsp90, heat shock protein 90; Sufu, Suppressor of fused.

Several CDK11 interacting protein partners have been identified in the transcription and RNA processing by implementing yeast two-hybrid interactive screens, tandem affinity purification, mass spectrometry analyses, immunoblot, and immunoprecipitation (Figure 2, Table 3). The first noteworthy finding is that cyclins L1 and L2 have been well documented as critical cyclin partner proteins of CDK11, which is necessary for the activation of the CDK11^p110^ protein isoform [61]. In addition, CDK11 also interacts with splicing factors, such as RNA-binding protein (RNPS1) and 9G8 [60, 65], as well as multiple transcriptional initiation and elongation factors, including RNA polymerase elongation factor 2 (ELL2), transcription factor IIF (TFIIF), transcription factor IIS (TFIIS), and facilitates chromatin transcription (FACT) [45]. RNA binding motif protein 15 B (RBM15B/OTT3) has been shown to interact with CDK11 as a competitor and antagonizes the positive effect of CDK11 in splicing [68]. CDK11 exerts the biological functions in cell cycle and apoptosis by interacting with several protein partners, including 14-3-3 protein and heat shock protein 70/90 (Hsp70/90) [93, 94].

**Table 3:** Characteristics of CDK11 interactiving partners

Interacting Partner	Subcellular Localization	Cellular Functions	Cancer Association	References
**CHK2**	nucleus	responses to genotoxic stress	mutation or low expression in breast, prostate, ovarian, colon, kidney, thyriod, bladder, lung cancer as well as sarcomas and leukemias	67
**CK2**	nucleus	promotes cell proliferation and growth, suppresses cell apoptosis	overexpression or hyperactivation in lung, breast, prostate, gastric, kidney cancer as well as AML, CLL and lymphomas	81; 90
**cyclin L1**	nucleus	regulates transcription and RNA splicing	overexpressed in human head and neck cancer, and is associated with lymph node metastases, also amplify in uterine cervical carcinoma and associates with poor prognosis	59; 61; 88
**cyclin L2**	nucleus	functions as a regulator of the pre-mRNA splicing process, modulates the expression of apoptotic and antiapoptotic proteins	overexpression of cyclin L2 inhibits cancer cell growth, induces apoptosis and cell cycle arrest, and enhances chemosensitivity	59; 61; 89
**cyclin D3**	nucleus and cytoplasm	forms a complex with CDK4 or CDK6, phosphorylates and inhibits RB protein and regulates the cell cycle G1/S transition	overexpressed and associated with a worse prognosis in patients with malignant melanoma, breast cancer, and non-Hodgkin's lymphoma	78
**RNPS1**	nucleus	regulates RNA splicing, promotes mRNA nuclear export and translation, as well as maintenance of postsplicing surveillance	overexpressed in mouse submandibular gland adenocarcinoma	60; 65
**9G8**	nucleus and cytoplasm	regulates RNA splicing, promotes nucleocytoplasmic export of mRNA and translate	not confirmed	60
**ELL2**	nucleus	promotes transcription initiation and elongation, directs immunoglobulin secretion, inhibits cell growth and survival, induces cell cycle arrest and apoptosis	increased expression in leukemia, mutation associated with multiple myeloma and salivary gland carcinoma	45
**TFIIF**	nucleus and cytoplasm	promotes transcription elongation by interacting with RNAPII throughout the elongation phase	unknown	45
**TFIIS**	nucleus and cytoplasm	promotes transcription elongation by enhancing the intrinsic endonucleolytic cleavage activity of RNAPII	unknown	45
**FACT**	nucleus	modulates nucleosome stability and chromatin remodeling, promotes DNA replication, recombination, and repair, as well as transcript elongation	overexpressed in breast carcinoma, non-small-cell lung cancer, renal cell carcinoma, and prostatic, pancreatic, and colorectal adenocarcinomas	45
**RBM15B/OTT3**	nucleus and cytoplasm	inhibits spliceosomal E complex formation, regulates RNA splicing and mRNA export	unknown	68
**14-3-3**	nucleus	regulates cell cycle, protein trafficking, and steroidogenesis, promotes cell proliferation, inhibits cell apoptosis	overexpressed in breast, lung, liver, head and neck cancers, as well as glioma and astrocytoma	94
**Hsp90**	cytoplasm and cell membrane	maintains normal tissue homeostasis	overexpression and/or hyperactivation in almost all human cancers	93
**Sufu**	nucleus and cytoplasm	promotes embryonic development	deletion, mutation or underexpression in lung, breast, prostate cancer, as well as in medulloblastoma	91; 92

Currently, there is no specific CDK11 inhibitor, which impedes the CDK11-targeted cancer therapy testing in preclinical models. However, the identification and characterization of these CDK11 interactors may facilitate the understanding of the mechanisms underlying CDK11 functions, as well as the development of therapeutic strategies for cancer. Indeed, some inhibitors targeting these CDK11 interactors have been successfully developed and used in preclinical evaluation or clinical trials, which may provide novel candidate compounds for cancer therapy. For example, CX-4945, a potent and selective small molecule inhibitor of CK2 and a confirmed CDK11 interacting protein, has been investigated for the treatment of prostate cancer [95]. Another small molecular compound, AZD7762, a CHK1/CHK2 (another validated CDK11 partner) inhibitor, has been evaluated in the treatment in patients with advanced solid tumors [96].

## EXPRESSION AND THERAPEUTIC POTENTIALS OF CDK11 IN DIFFERENT HUMAN CANCERS

Due to their critical roles in the growth and proliferation of many types of human cancer, CDKs comprise an attractive set of targets for novel anticancer drugs development. Many CDKs inhibitors are currently being assessed in preclinical or in clinical trial investigations for cancer therapy; among them, a CDK4/6 selective inhibitor palbociclib has recently been approved for breast cancer treatment due to its selected and promising inhibitory effect on ER+/HER2- breast cancer [15–18]. CDK11 function is a critical regulator of cell cycle progression and RNA transcription, and recent studies have suggested that CDK11 also plays important roles in several types of human cancers (Table 4).

**Table 4:** Functions of CDK11 in different human cancers

Human Cancers	Study Technique	Functions in Tumors
**Breast Cancer**	immunohistochemistry; RNAi; immunofluorescence assay; cell viability assay; cell colony formation assay; cell migration assay; western blot; flow cytometry analysis; xenograft RNAi studies	promotes breast cancer cell proliferation, growth, migration and cell cycle progression; inhibits breast cancer cell apoptosis, negatively correlated with breast cancer patient clinical prognosis
**Osteosarcoma**	systematic Kinome shRNA screening; CRISPR, RNAi; cell proliferation assay; apoptosis assay; western blot; immunofluorescence assay; immunohistochemistry; xenograft RNAi studies	promotes osteosarcoma cell proliferation, growth; inhibits osteosarcoma cell apoptosis; negatively correlated with osteosarcoma patient clinical prognosis
**Liposarcoma**	immunohistochemistry; RNAi; cell proliferation assay; western blot; immunofluorescence assay; chemotherapeutic response assay	promotes liposarcoma cell growth, survival; inhibits liposarcoma cell apoptosis; desensitizes liposarcoma cell to chemotherapy
**Multiple Myeloma**	high-throughput siRNA screening;RNA microarray hybridization	promotes myeloma cell proliferation and survival
**Colon Cancer**	high-throughput RNAi screening	acts as a positive modulator of the Wnt/β-catenin pathway in colon cancer
**Cervical Cancer**	RNAi; microtubule regrowth assays	promotes centrosome maturation and bipolar spindle morphogenesis in cervical cancer cells
**Acute Myeloid Leukemia (AML)**	CRISPR-based knockout with RNAi	crucial for TSC2-deficient AML cell growth

## CDK11 IN BREAST CANCER

Recently, the expression and association of CDK11 in human breast cancer has been explored [21]. Immunohistochemical and Western blot assay have revealed that CDK11 is highly expressed in both breast tumor tissues and cell lines. Elevated CDK11 expression in breast cancer tissues significantly correlates with poor differentiation, and is also associated with advanced TNM stage and poor clinical prognosis for breast cancer patients. *In vitro* knockdown of CDK11 by siRNA significantly inhibits cell growth and migration, and dramatically induces apoptosis in breast cancer cells. Similar results have also been found almost simultaneously by another investigation group, which also showed that CDK11 expression is essential for the maintenance of the aggressive characteristic of triple negative breast cancer (TNBC) [97]. Immunohistochemical analysis of TNBC patient tissues showed that 100% of tumors stained positive for CDK11 with high nuclear intensity compared to normal tissue. The Cancer Genome Atlas analysis by comparing basal to other breast cancer subtypes, and to normal breast tissues revealed statistically significant differences in CDK11 expression. Downregulation of CDK11 in breast cancer cells resulted in significant loss of cell viability and clonal survival, reduced CDK11 relevant mRNA and protein expression, and induced cell death changes [97]. Furthermore, *in vivo* treatment with tenfibgen-siCDK11 nanocapsules caused MDA-MB-231 xenograft tumor shrinkage, loss of proliferation, and decreased expression of targeted genes. These findings suggest that CDK11 is critical for the survival and proliferation of breast cancer cells, which highlight that CDK11 may be a promising target for therapeutic development in breast cancer.

## CDK11 IN MULTIPLE MYELOMA

A kinase-wide RNAi lethality study using high-through kinome siRNA screening identified CDK11 as a vulnerable kinase in human multiple myeloma, which proposes that inhibition of CDK11 represents a uniquely targeted novel therapeutic strategy in human multiple myeloma [22]. To better define potential drug targets in myeloma disease, another investigation conducted a focused RNAi lethality screening and consistently found that CDK11 is a crucial survival gene in multiple myeloma [98]. To further assess CDK11 for myeloma-selective vulnerability, the study evaluated the expression of CDK11 in human multiple myeloma, and showed that CDK11 is significantly upregulated in expression in primary multiple myeloma tissues, as compared with normal human primary tissues. Collectively, CDK11 is overexpressed and functions as a critical survival gene in human multiple myeloma, which suggests that CDK11 may represent a promising druggable target for human multiple myeloma therapy.

## CDK11 IN OSTEOSARCOMA

Osteosarcoma is the most common primary malignant tumor of bone. A kinase shRNA screening first identified CDK11 as essential for the survival of osteosarcoma cells [19]. Osteosarcoma cells display high levels of CDK11 expression, and CDK11 knockdown inhibited cell growth and induced apoptosis in osteosarcoma cells [19]. Moreover, immunohistochemical analysis showed that osteosarcoma patients with high CDK11 expression are associated with significantly shorter survival than patients with low CDK11 expression. Systemic *in vivo* administration of *in vivo* ready siRNA of CDK11 reduced tumor growth in an osteosarcoma subcutaneous xenograft model. Furthermore, a recent study using a novel robust and highly efficient genome editing tool, the clustered regularly interspaced short palindromic repeats-Cas9 (CRISPR-Cas9) system, to silence endogenous CDK11 DNA found that CDK11 knockout significantly reduced osteosarcoma cell viability, proliferation, migration, invasion, and induced cell death [99]. All these results show that CDK11 signaling is essential in osteosarcoma cell growth and survival, further elucidating the regulatory mechanisms controlling the expression of CDK11 and ultimately indicate that developing a CDK11 inhibitor may provide therapeutic benefit against osteosarcoma.

## CDK11 IN LIPOSARCOMA

Similarly, the functional and therapeutic relevance of CDK11 as a putative target in liposarcoma, another type of mesenchymal tissue-originated malignancy, was also studied. Immunohistochemical analysis of liposarcoma tissue microarray (TMA) showed that CDK11 is highly expressed in liposarcoma tissues as compared with benign lipoma tissues. CDK11 knockdown by synthetic siRNA or lentiviral shRNA decreased cell proliferation and induced apoptosis in liposarcoma cells. Moreover, CDK11 knockdown enhanced the cytotoxic effect of doxorubicin in liposarcoma cells [20]. These results suggest that CDK11 may be a promising therapeutic target for liposarcoma treatment. Future studies on the signaling pathway and molecular mechanisms of CDK11 and cell growth in liposarcoma are required.

## CDK11 IN OTHER TYPE OF HUMAN CANCERS

The pioneer studies has also confirmed that CDK11 is ubiquitously expressed many human cancer cell lines, such as Jurkat, Cem C7, HeLa, HEK 293, K562, suggesting the potential functional roles of CDK11 in carcinogenesis [40, 100]. Furthermore, an unbiased high-throughput RNAi screening found that CDK11 is a positive modulator of the Wnt/β-catenin pathway in colon cancer [84]. In cervical cancer, CDK11 knockdown by RNAi in HeLa cells induced abnormal spindle assembly, mitotic arrest by checkpoint activation, and cell death [71]. More recently, by using novel CRISPR-based knockouts with RNAi technology, CDK11 has been identified is the crucial gene in cells lacking either the tuberous sclerosis complex 1 (TSC1) or TSC2, but not that of wild-type cells. Mutation or aberrant inhibition of the TSC complex is common in various human tumor cancers. Knockdown of CDK11 showed growth-inhibiting effects in mammalian TSC2-deficient cell lines, including human tumor-derived (AML) cells. TSC1 and TSC2, function together in an evolutionarily conserved protein complex that is a point of convergence for major cell signaling pathways that regulate mTOR complex 1 (mTORC1) [101].

Taken together, CDK11 is usually overexpressed and/or activated in several types of human malignancies, and the overexpression and/or hyperactivation is highly associated with poor outcomes in cancer patients. Consequently, CDK11 may present as a selective and specific target for cancer therapy. The downregulation of expression and activity of CDK11 may be a novel therapeutic modality in cancer treatment.

## CDK11 IN OTHER HUMAN DISEASES

In addition to human cancer, CDKs play important roles in other types of human diseases. Among them, CDK7, CDK9 and CDK13 have been found to be critical for HIV replication inside cells. However, the function of CDK11 remains largely unknown. Recently, CDK11 has been found to regulate the cleavage and polyadenylation (CPA) of all viral transcripts [102, 103]. CDK11 was found to be associated with the TREX/THOC, which recruited this kinase to DNA. Once at the viral genome, CDK11 phosphorylated serines at position 2 in the CTD of RNAP II, which increased levels of CPA factors at the HIV 3′ end. Higher levels of CDK11 increased the length of HIV poly(A) tails and the stability of mature viral transcripts. These data suggests that CDK11 also plays an important role in the transcriptional regulation of HIV mRNA.

## CONCLUSIONS

Significant evidences have shown that overexpression and hyperactivation of CDKs are common in cancers, and targeting CDK has emerged as a highly promising therapeutic strategy. Specifically, overexpression of CDK11 has been shown to be associated with different cancers recently. As CDK11 has been found to play a crucial role in cancer cell proliferation and growth, CDK11 represents a promising target for novel anti-cancer drug development.
